# Meningitis as an Initial Presentation of COVID-19: A Case Report

**DOI:** 10.3389/fpubh.2020.00474

**Published:** 2020-09-22

**Authors:** Sidra Naz, Muhammad Hanif, Muhammad Adnan Haider, Mukarram Jamat Ali, Muhammad Umer Ahmed, Sana Saleem

**Affiliations:** ^1^Department of Internal Medicine, University of Health Sciences, Lahore, Pakistan; ^2^Khyber Medical College Peshawar, Hayatabad Medical Complex, Peshawar, Pakistan; ^3^Department of Internal Medicine, Allama Iqbal Medical College, Lahore, Pakistan; ^4^Department of Internal Medicine, King Edward Medical University Lahore, Lahore, Pakistan; ^5^Ziauddin University and Hospital, Ziauddin Medical College, Karachi, Pakistan

**Keywords:** SARS-CoV-2, COVID-19, meningitis, meningo encephalitis, neurological manifestation

## Abstract

The common presenting symptoms of fever, fatigue, and mild respiratory symptoms like dry cough, are associated with COVID-19, however, patients can also develop neurological manifestations like headache, anosmia, hyposmia, dysgeusia, meningitis, encephalitis, and acute cerebrovascular accidents during the disease. Although very rare, these neurological manifestations are sometimes the sole initial presenting complaint of COVID-19. This case report discusses patients where the initial presenting symptoms seemed to be exclusive to meningitis but the later diagnosis was COVID-19. It is important to increase awareness of these rare presentations in physicians and healthcare workers and facilitate early diagnosis and management to prevent the horizontal spread of the disease.

## Introduction

Following its emergence in Wuhan, China in December 2019, the Coronavirus disease 2019 (COVID-19), which is caused by severe acute respiratory coronavirus-2 (SARS-CoV-2), has become a pandemic (1) and been declared a global health emergency (2). In addition to the common presenting symptoms of fever, fatigue, and mild respiratory symptoms like dry cough, patients with COVID-19 can also develop neurological manifestations like headache, anosmia, hyposmia, dysgeusia, meningitis, encephalitis, and acute cerebrovascular accidents during the course of the disease (3, 4). The first case of meningitis associated with COVID-19 was reported in Japan in February 2020 (5). Since then, two or three more cases of meningoencephalitis have also been reported in the United States (6–8). Although very rare, these neurological manifestations sometimes are the sole initial presenting complaint of COVID-19. In this article, we present a case discussion of instances in which the initial presenting symptoms were exclusive to meningitis and later diagnosed as COVID-19, to make physicians and healthcare workers cognizant of such rare presentations. It is important to diagnose and manage these patients at the earliest possible stage of treatment to prevent the horizontal spread of COVID-19.

## Case Report

A 21-years-old male medical student with no known co-morbidities was presented to an emergency department with a 2-days history of frontal headache and fever, and 1-day history of neck stiffness. He denied any cough, shortness of breath, body aches, and diarrhea (Table 1). On physical examination, he was alert, oriented, and awake with a Glasgow coma scale score of 15/15. He had a fever of 101 F and neck rigidity with absent Babinski sign and 2+ deep tendon reflexes.

**Table 1:** Demographics and clinical characteristics.

**Characteristics**	**Patient**
Age	21
Sex	Male
Significant past medical history	None
Symptoms onset	Frontal headache, Fever, and neck stiffness
Respiratory distress	Developed 5 days after symptoms onset
Cause of death	Multi-organ failure

Based on clinical presentations and initial blood work up, bacterial meningitis was suspected and he was started on intravenous antibiotics empirically after cerebral spinal fluid (CSF) was sent for analysis. CSF analysis showed a picture of viral meningitis and in addition to empiric antibiotics, he was also given antiviral agents. CSF gram staining, Ziehl-Neelsen staining, and culture showed no microorganisms, and tests for Herpes simplex type 1, Herpes simplex type 2, and Varicella zoster virus were negative (Table 2).

**Table 2:** Cerebrospinal fluid analysis.

**Tests**	**Results**
Appearance	Clear with no xanthochromia
Lactate dehydrogenase	48 U/L
Glucose	83 mg/dL
Protein	164 mg/dL
RBCs	05
Neutrophils	10
Lymphocytes	90
Gram stain/Ziehl-Neelsen Stain	No micro-organisms seen
HSV-PCR	Negative
VZV-PCR	Negative
Culture	No growth after 48 h of incubation

**Figure 1:**
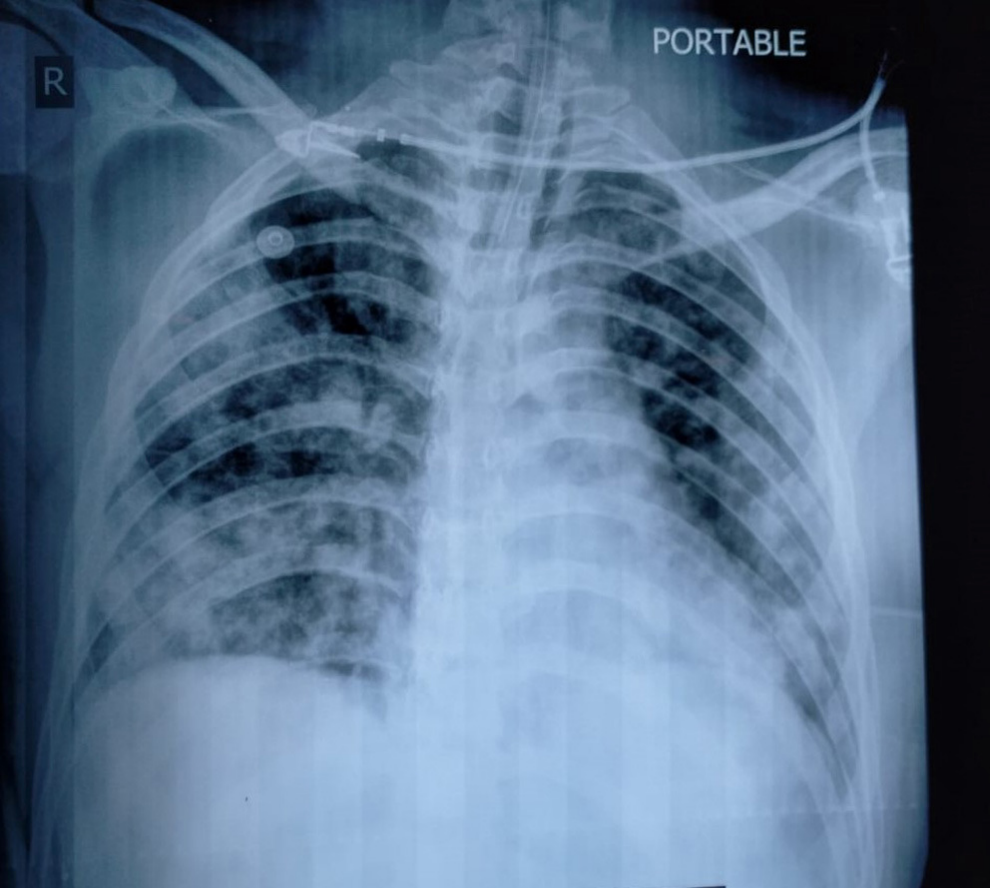
Chest X-ray shows diffuse multi-lobar infiltrates consistent with acute respiratory distress syndrome.

On day 2 of hospitalization (day 4 of initial symptoms), he had swelling of his left eye, and a computed tomography (CT) head was ordered on neurologist recommendation which showed no significant findings. Even though he had no respiratory symptoms of cough and shortness of breath, a chest x-ray was ordered due to the ongoing COVID-19 pandemic and it showed a patch of consolidation. Based on these X-ray findings, testing for COVID-19 was done and a reverse transcriptase polymerase chain reaction for SARS-CoV-2 on nasopharyngeal swab was positive on day 5 of hospitalization. On that same day, he developed tachycardia, tachypnea, and hypotension; his oxygen saturation started to drop progressively and was put on a ventilator. His chest X-ray showed diffuse multi-lobar infiltrates consistent with acute respiratory distress syndrome (Figure 1). His laboratory work up showed respiratory acidosis and a picture of disseminated intravascular coagulation (DIC) (Table 3). One day later, he passed away due to multi-organ failure.

**Table 3:** Laboratory findings (on day 5 of initial symptoms).

**Test**	**Results**
Leukocytes count	2.2 (x10^9^/l)
Lymphoctes	07%
Neutrophils	89%
Platelet count	65 (x10^9^/l)
Serum procalcitonin	50 ng/mL
Serum ferritin	1,358 ng/mL
Serum CRP	>32 mg/dL
Serum LDH	527 U/L
Serum CK-MB	54 U/L
Albumin/globulin ratio	0.8
Serum AST	88 U/L
Serum albumin	2.6 g/dL
Serum total protein	5.7 g/dL
International normalized ratio	1.7
Protheombin time	18 s
Plasma FDPs	8,340 ng/FEUm
pH	7.295
HCO_3_	22.3 mmol/L
PCO_2_	52 mmHg
Base excess/deficit	−2.7 mmol/L

## Discussion

This case indicates that in addition to common presenting symptoms of fever, fatigue, and mild respiratory symptoms like dry cough and shortness of breath, patients with COVID-19 can also develop neurological manifestations like headache, anosmia, hyposmia, dysgeusia, meningitis, encephalitis, and acute cerebrovascular accidents during the course of the disease (3), which highlights the neurotropic potential of SARS-CoV-2 (4). To date, the underlying pathophysiological mechanisms through which SARS-CoV-2 implicates the central nervous system (CNS) are not fully understood, however, the following mechanism have been proposed in other studies (9, 10):

1. Direct spread of SARS-CoV-2 to brain.
2. Spread through neuronal pathways.
3. Haematogenous spread to brain.
4. Immune mediated injury (cytokine storm syndrome).
5. Hypoxic related injury to CNS.

In this case, the patient initially presented with fever and frontal headache along with neck stiffness. There was a delay in the diagnosis because this initial presentation of patients with COVID-19 is rare and only a few cases have been reported so far. To the best of our knowledge, in the USA only two cases have been reported where the initial presenting complaint was a meningitis-like illness (6, 8).

The RBCs in the CSF are an indication of blood brain barrier breach which can occur in SARS-CoV-2 and has been linked to cytokine storm syndrome. Cytokine storm syndrome related damage to the central nervous system has also been implicated in many other viral infections (11). However, only five RBCs in the CSF of our patient could be attributed to traumatic lumbar puncture. Likewise, it has also been suggested that cytokine storm syndrome causes severe symptoms and brain damage in patients with COVID-19 and this is supported by the fact that patients having severe symptoms associated with SARS-CoV-2 infection respond to interleukin-6 (IL-6) receptor blocker [i.e., tocilizumab (12)]. Together with typical picture of viral meningitis on CSF analysis, negative polymerase chain reaction for herpes simplex, and positive reverse transcriptase polymerase chain reaction for SARS-CoV-2 on a nasopharyngeal swab, we labeled this case of meningitis as viral meningitis secondary to SARS-CoV-2. Although there have been some cases reported of SARS-CoV-2 detection in CSF by reverse transcriptase polymerase chain reaction (RT-PCR) (5, 13), yet US Food and Drug Administration has not approved any test to detect SARS-CoV-2 in CSF. Additionally, as the CT head was normal in this case, we should have ordered a magnetic resonance imaging of the brain for detailed imaging.

During this ongoing pandemic, there is a need to make physicians and other healthcare workers cognizant of rare presentations such as this, so that we can diagnose and manage these patients at the earliest possible opportunity, which prevents the horizontal spread of the virus and ensures patient safety. We recommend testing of CSF for SARS-CoV-2 *via* RT-PCR in suspected cases.

## Data Availability Statement

The raw data supporting the conclusions of this article will be made available by the authors, without undue reservation.

## Ethics Statement

Written informed consent was obtained from the individual(s) for the publication of any potentially identifiable images or data included in this article.

## Author Contributions

All authors listed have made a substantial, direct and intellectual contribution to the work, and approved it for publication.

## Conflict of Interest

The authors declare that the research was conducted in the absence of any commercial or financial relationships that could be construed as a potential conflict of interest.
